# Analysis of hepatocellular carcinoma associated with hepatitis B virus

**DOI:** 10.1111/jcmm.17867

**Published:** 2023-07-30

**Authors:** Litao Zheng

**Affiliations:** ^1^ Clifton College Bristol UK

**Keywords:** cancer, hepatitis B virus, hepatocellular carcinoma, infection, malignancy

## Abstract

The hepatitis B virus (HBV) is considered one of the main driving forces in the development of hepatocellular carcinoma (HCC). Human HBV is a partially double‐stranded DNA (dsDNA) virus consisting of approximately 3.2 kbp. HBV predominantly infects hepatocytes via the receptor sodium taurocholate cotransporting polypeptide (NTCP) and coreceptor hepatic proteoglycan. The replication of HBV in hepatocytes leads to apoptosis while simultaneously leading to cirrhosis and cancer. Although the integration of dsDNA into the hepatocyte genome seems to be the main cause of mutation, since the discovery of their function, viral proteins have been shown to regulate the P53 pathway or P13K/AKT pathway to prevent host cell apoptosis, causing uncontrolled proliferation of liver cells leading to the formation of solid tumours. The most common treatments involve nucleo(s)tide analogue (NA) and polyethylene glycol (PEG)ylated interferon‐alpha (PegIFN‐α). NA treatment has been found to be effective for the majority of patients and induces few side effects. Nevertheless, the rate of seroconversion is relatively low. PegIFN treatment is contraindicated during pregnancy and leads to a higher morbidity rate, but the seroconversion rate is high. Since medicines and vaccines have been developed, the incidence and mortality of HBV related to HCC have profoundly decreased compared to those in 2000. This review investigates what can be the potential mechanism that HBV can cause HBV and the treatment used in chronic and acute infection.

## 1 INTRODUCTION

Hepatocellular carcinoma (HCC) is considered the most progressive and common carcinoma worldwide.^1^

The consequences of HCC rank it among the deadliest among all types of carcinomas. The number of new cases of liver cancer reached approximately 840,000 worldwide, with 780,000 deaths reported, in 2018; in the United Kingdom, approximately 6000 people are diagnosed with HCC each year.^2^ The incidence and mortality have increased rapidly in recent decades, increasing from 1.8 to 5.5 cases per 100,000 persons, and it is projected to be cancer with the highest average rate of increase in the next 15 years.^3^, ^4^

Chronic hepatitis B virus (HBV) infection is one of many HCC aetiologies. The prevalence of chronic HBV infection in the United Kingdom was estimated to be approximately 1.1%, which remains relatively low by international standards.^5^ The prevalence of the disease in the total UK population is 3.5%, and 257 million people in the UK live with HBV.^6^ In August 2017, the UK childhood immunization programme started offering the HBV vaccine to children who are at high risk of infection and those with liver disease. The immunization programme helped protect children against future exposure to the effect of the virus and reduced the prevalence of HBV.^7^

Approximately 296 million people are infected with HBV globally. In western Pacific regions, which are highly populated, HBV prevalence is approximately 116 million people.^1^ The main challenge that the WHO faces in a respective region is the reduction in vertical transmission (e.g. mother‐to‐child transmission), which is the primary route of HBV infection. Unsterilised medical equipment is another cause. In the United Kingdom, the majority of people who have acquired HBV are from endemic countries, increasing the spread throughout the United Kingdom (UK health security agency 2023).^8^ The routes of increasing HBV transmission involve bodily fluid exposure and non‐medical drug injection with shared needles (WHO, 2023).^9^

According to Burton et al.,^3^ HBV prevention involves reducing needle sharing among people who inject drugs (PWID) and increasing the vaccination of healthcare staff. This has led to a significant decrease in the incidence and mortality among both men and women.^3^

Hepatitis B virus is a partially double‐stranded DNA (dsDNA) virus that belongs to the family Hepadnaviridae.^10^ However, HBV mainly infects hepatocytes, increasing the risk of developing HCC through various mechanisms. There are mainly two methods of treatment. The primary treatment consists of nucleos(t)ide analogues (NA) and immunomodulators, such as polyethylene glycol (PEG)ylated INFa.^3^ In patients receiving NAs, the cumulative virological response reaches 97%, and PegIFNa leads to seroconversion, with anti‐HBe antibody production of 32% in patients (Bamford and Zuckerman, 2021).^11^ Corresponding to 48–60 million global infections, 13.02% of chronic HBV‐infected individuals have been found to be coinfected with hepatitis D virus (HDV).^12^ As a result, the true prevalence of HBV‐only infection remains unknown in the United Kingdom and in the world.

**FIGURE 1:**
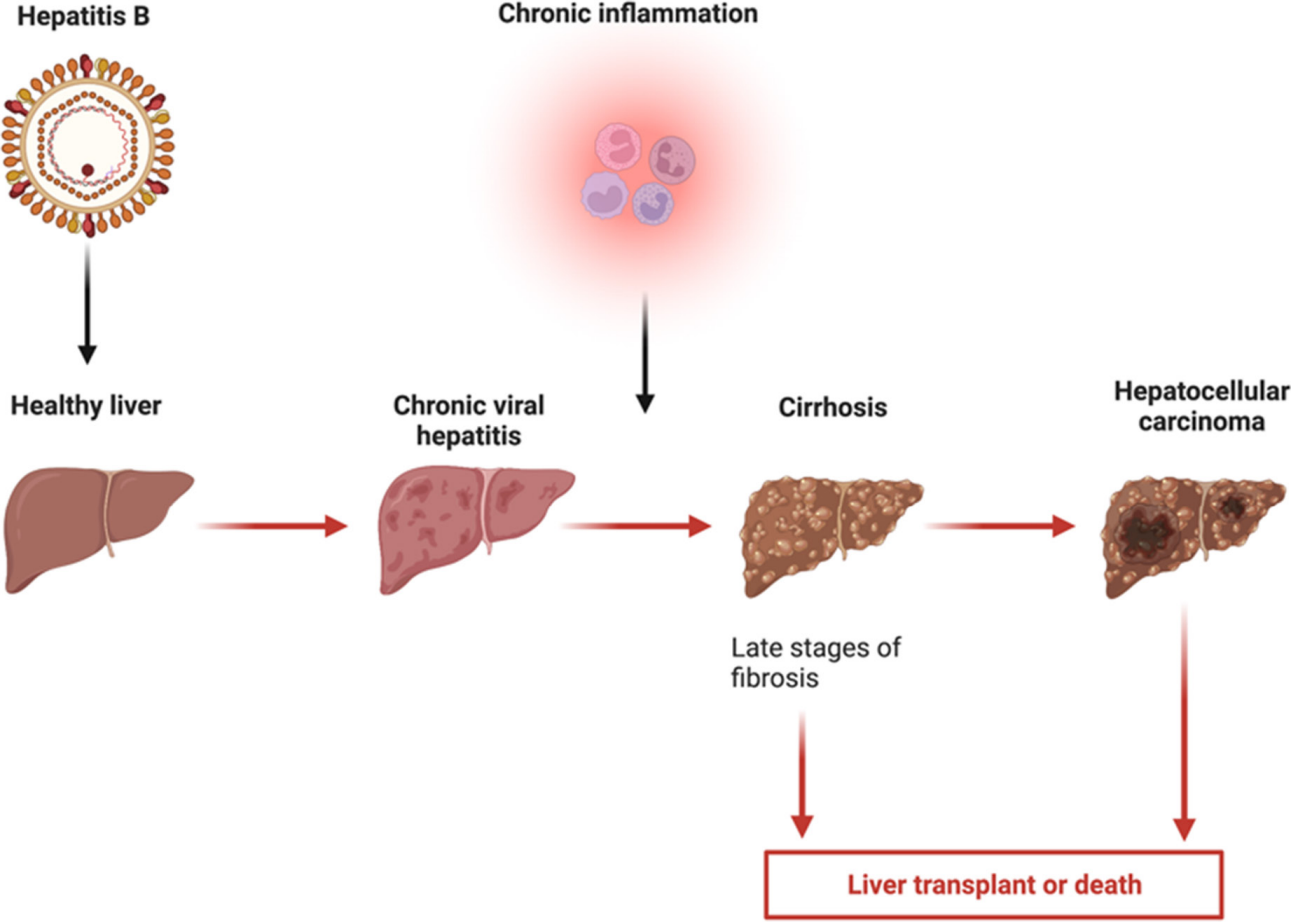
An overview of the pathogenesis of infection‐induced hepatocellular carcinoma. The figure was created using Biorender.

Most studies investigating the connection between HCC and HBV have proven a correlation (Figure 1).

Elevated HBV marker levels in the serum and tissue of patients with HCC were the first direct evidence to prove an association between chronic HBV infection and HCC.^13^ The level of hepatitis B surface antigen (HBsAg) in patients with HCC is typically higher than that in patients with either cirrhosis or chronic hepatitis.^13^

According to Peters and Locarnini,^14^ HBsAg can be detected in the serum in as many as 85% of HCC patients compared with 15% or fewer in control subjects in populations with the highest incidences of HCC and where chronic HBV infection is endemic or hyperendemic. This evidence proves that carcinogenesis induced by HBV is linked with viral surface proteins. However, the complete mechanism by which HBV causes HCC remains unclear. Direct evidence includes the integration of the HBV genome into the hepatocytes.

This paper discusses the relationships between HBV and HCC while simultaneously describing the mechanisms by which HBV infection may cause HCC, the prevalence of HBV in the United Kingdom, and the available treatments and prevention programmes directed to reduce the incidence of HBV‐related HCC.

## 2 METHODS

The data available at PubMed and Elsevier were searched from inception to February 2023 for peer‐reviewed articles in English. They searched terms such as “prevalence,” “Hepatitis B virus,” “hepatocellular carcinoma,” “HBV viral entry mechanism,” “PegINF‐α,” “Tenofavir,” “Nucleotides or Nucleotides analogue” and “PegIFN‐α treatment.” The articles and the titles were read for suitability, and necessary information was extracted. Publicly available search engines such as Google were searched with similar terms for additional resources. The figure was created using Biorender.

## 3 DISCUSSION

### 3.1 HBV related to HCC

The HBV genome consists partially of dsDNA or relaxed circular molecule (rcDNA). With 3.2 kb, it is the smallest DNA virus discovered to date. The negative sense strand (−) completely overlaps the incomplete positive sense strand (+), with approximately 600 fewer nucleotides than the negative strand.^15^ It is one of the most compact genomes, as it contains four wholly open reading frames (ORFs), and no termination site has been identified.^16^ This genome incorporates all types of regulatory signals, such as enhancers. Among these enhancers, Enh1, which is upstream of the x promoter, is targeted by various basic activators, such as leucine zipper proteins, and Enh2, which is upstream of the pre‐S promoter, is primarily targeted by nuclear receptors (Nrs). Pre‐S1/Pre‐S2 and S are promoters and encode HBsAg.^16^ Gene X encodes the protein HBx, a non‐structural protein mainly involved in hepatic disease; however, the specific mechanism of HBx action remains unclear. Furthermore, Pre‐C and C encode a secondary protein core protein, HBV e antigen (HBeAg), which is necessarily measured to determine whether a patient is presenting with chronic hepatitis.^16^

The translation of the viral S gene leads to the production of small (S), medium (M), and large (L) surface proteins; that is, M and L are longer than the S protein, where the L protein harbours Pre‐S1, Pre‐S2 and S region.^17^ HBsAg is an integral membrane protein that is also glycosylated. Among the three translated S transcripts, S is the most abundant protein. Notably, the N‐terminus of the pre‐S1 region in the L protein is critical for the interaction with the hepatic receptor.^11^ However, the pre‐S1 region in the L protein has been shown to interact with the capsid of the virus and have a mucogenic effect. This is because during maturation of the viral particles, 50% of the pre‐S domain translocates from the cytoplasmic region to extracellular space.^18^ In addition to glycosylation, L HBsAg undergoes myristoylation. This modification can increase the affinity of binding of the L protein with the NCTP receptor and host cell envelopment of viral particles.^19^

Two mRNA strands are longer than the viral genome (3.5 kb). The precursor HBeAg protein is often referred to as the pre‐core protein. The core protein is formed during the second round of translation (HBcAg).^20^ The HBcAg is approximately 185 amino acids (aa) long. This aa sequence can be divided into two parts: (1) The N‐terminus, comprising approximately 135 aa, is called the self‐assembly domain, and (2) the C‐terminus, comprising 35 aa, is a positively charged domain enriched with arginine residues. The positive charge is essential for genome packaging.^20^ Therefore, one therapeutic approach to control viral replication involves disrupting the formation of nucleocapsids. Heteroaryl dihydropyrimidine (HAP) Bay 41‐4109, which was discovered in 2009, misdirects the assembly of the nucleocapsid, leading to a reduction in viral replication and hence a reduction in viral load.^20^

The HBx protein is translated by the shortest transcripts, such as that 0.7 kb in length, and comprises 154 aa.^19^ This protein has been suggested to be involved in the regulation of gene expression in primary hepatocytes. However, recent studies have suggested that the expression of HBx is not a direct cause of HCC; in contrast, it interferes with several regulatory factors during transcription.^21^ For example, HBx inactivates the p53 protein and disrupts the DNA repair system. The carboxyl terminus of p53 is a binding site for HBx. P53 protein binding to HBx leads to p53 migration from the nucleus to the cytosol. P53 in the cytosol ultimately causes uncontrolled cell cycle progression and division, which can then be considered a cause of HCC. Interestingly, the expression of HBx can regulate HBV replication and the survival of hepatocytes by PI3K/AKT pathway^22^ (Table 1).

**TABLE 1:** An overview of different proteins involved with HBV.

Protein	Function	Reference
HBsAg	The S protein interacts with low‐affinity HSPG receptor, and L protein interacts with high‐affinity NTCP. This leads to endocytosis of the virus	Schädler and Hildt^20^
The core protein	Is essential for genomic packaging	Schädler and Hildt^20^
HBX protein	It interferes with several regulatory factors during transcription. For example, HBx inactivates the p53 protein and disrupts the DNA repair system	Ali et al.^21^

Abbreviations: HSPG, heparan sulphate proteoglycan; HBsAg, hepatitis B surface antigen; HBV, hepatitis B virus; NTCP, sodium taurocholate cotransporting peptide.

### 3.2 The life cycle of HBV in HCC

The attachment of a virus to a cell is a multiple‐step process that, in hepatocytes, involves the basolateral membrane. The first step involves an energy‐independent low‐affinity interaction between the virus and the cell.^20^ The low‐affinity receptor heparan sulphate proteoglycan (HSPG) subsequently interacts with the pre‐S1 domain of SHBsAg or the N‐terminus of HBsAg. This binding is proceeded by the electrostatic attraction between the negatively charged HSPG and two positively charged residues on the S domain. And then to a high‐affinity binding protein, namely, the bile salt transporter sodium taurocholate cotransporting peptide (NTCP). This high‐affinity binding shows significant importance for the infectivity of HBV. Research found that 11 amino acid deletion of the pre S 1 domain increases the affinity of binding to the NCTP. After the attachment, the virus enters the cell via clathrin‐mediated endocytosis predominantly. However, the receptor other than NCTP is still unknown. According to the viral entry of hepatitis C virus (HCV), the viral entry involves at least 14 host cell factors to enhance the infectivity of HCV. Research shows that the efficiency of the infection remains low in cell lines that are over‐expressing NTCP. The internalization of HBV recently being suggested is through clathrin‐mediated endocytosis. This mechanism was supported by the evidence that a high level of adapting factor 2 being observed and also inhibiting the production of the heavy chain of the clathrin protein further shows the decrease in the infectivity of HBV. Furthermore, the main mechanism of NCTP taking up bile salt is through a clathrin‐mediated pathway; therefore, HBV is more likely to enter the cell by this process.

Once the virus enters the cell, it forms an early endosome. HBV uses mucogenic mechanism to escape from the degradation by lysosomes. HBV achieve this by using the fusogenic region identified in the L HBsAg (Liu, 2016).^23^ This includes the C terminal half of the pre‐S‐2 region, which is pH‐independent, also involves the N terminal part of the S region, which is low pH dependent and finally, the pre‐S‐1 region, which is pH dependent (Liu, 2016).^23^ The dominant fusogenic domain is identified using lipid mixing assays. HBV used for lipid mixing assays which lack pre‐1 domain seems to lose all the fusogenic function. This supports the point that the Pre S‐1 region is mainly used for fusion in low pH conditions (Liu, 2016).^23^

pgRNA consists of a capped 5′ end that incorporates ε, forming a secondary loop structure. Specifically, a lower stem‐loop is formed by an internal bulge that separates the strands downstream of the apical loop. pgRNA harbours DR1 and DR2, and a secondary structure is formed at the 3′ end (Hu, 2009).^24^, ^25^ The cap on the 5′ end plays an important role in the initiation of negative DNA strand synthesis. The following steps are realized within a nucleocapsid. First, the polymerase recognizes the pgRNA secondary structure, which triggers the synthesis of a 4‐nucleotide DNA primer, which is translocated from the 5′ end to the 3′ end, where a primer has hybridized with DR1. This movement is facilitated by two elements, namely, Φ and ω at the 3′ end, which interact with ε and form a loop.^3^ Through negative‐strand synthesis, the RNase domain of the polymerase degrades pgRNA, except for the final 10–16 nucleotides, leaving a capped RNA oligomer. Since the polymerase is covalently attached to the 5′ end, a loop is formed. Finally, the remaining nucleotides are translocated to DR2, which primes the synthesis of the incomplete positive strand.^3^ Ten percent of oligonucleotides are translocated to DR1 and prime dslDNA synthesis, whereas 90% of oligonucleotides are translocated to the DR2 region and prime the synthesis of rcDNA.^26^

Exposure to HBV increases the probability of an individual developing chronic hepatitis. The severity of the infection depends on the level of HBV‐DNA expressed. High levels of HBV DNA (>2000 IU/mL) suggest that the concentrations of viral proteins, such as HBsAg, HBeAg and HBx, are also high. The aforementioned proteins are the main regulatory proteins of host gene expression and cell cycle progression in hepatocytes, and their regulatory effects can eventually lead to HCC.^27^

Recent studies have indicated that HBV is critical for 30% of cases with cirrhosis and 50% of cases with HCC, which has become the second carcinogen ranked after tobacco.^3^ The causation of HCC related to HBV can be either direct or indirect. The direct cause of HCC is the integration of viral DNA into hepatocyte DNA in turn to disrupt its regulatory gene; however, the integration site seems to be random. On the other hand, the indirect cause of HCC is preceded by a protein that inactivates regulatory proteins such as p53 and hijacks certain transcription factors. Moreover, HBV activates various signalling pathways, such as apoptosis, which ultimately leads to tumour formation.

### 3.3 The integration of HBV DNA into the host genome and its role

As mentioned above, 10% of pgRNA is synthesized as double‐stranded linear DNA (dslDNA), which is then integrated into the host cell genome. dslDNA encodes HBX, pre‐S/S, polymerase and precore/core proteins. Moreover, dslDNA can be converted into wild‐type cccDNA, probably via recombination. This conversion replenishes the intracellular HBV cccDNA pool in a host cell nucleus and thus upregulates the synthesis of target proteins and increases viral load. The Enhancer 1 domain in dslDNA is the promoter of the HBx gene. However, truncation of the sequence that encodes the HBx C‐terminus does not affect HBx function in transactivated transcription. The lack of stop codons leads to the production of the HBV–cell fusion transcript HBx–long interspersed nuclear element 1 (LINE1). Studies have revealed a close relationship between HBx–LINE1 fusion proteins and HCC tumourigenesis.^28^

The research on the role is primarily focused on the carcinogenesis of the liver that derives HCC. The mechanism includes (1) *cis*‐mediated insertional mutagenesis of HCC‐associated genes and (2) induction of chromosomal instability by integrated DNA, which are mostly understood. Research on woodchuck HBV (WHBV) suggests that there is a high rate of DNA insertion detected in chronic infection, which leads to the development of HCC in woodchuck. However, human HBV associated with HCC remains unclear. Recent studies have used next‐generation sequencing techniques to search for integration site specificity between tumour and non‐tumour tissues. The results indicate that both coding and promoter regions express high integration rates. Nevertheless, this is not shown in all studies; therefore, it is unclear whether it has direct effects on the development of HCC.^26^

Furthermore, integration in the LINE1 gene results in the formation of fusion transcripts.

Fusion transcripts are non‐coding RNA transcripts that can activate the WNT/B signalling pathway. This leads to the upregulation of β‐catenin and cell division and the cell cycle, thereby promoting HCC.^26^ Most of the integration being observed is in the repeated site, CpG island and telomeres compared to non‐tumour cells. The S/MAR region, which is observed in both the WHB and human HBV, promotes HCC progression.^26^

HBx is a 154 aa protein that causes the development of HCC due to its regulatory function in gene expression and activation of certain signalling pathways. However, HBx is not the direct driving force behind HCC tumorigenesis. In a transgenic mouse model of fibrosis, HBx promoted cell cycle progression in fibroblasts by increasing the expression of transforming‐growth factor β1 (TGF‐β1) and connective tissue growth factor (CTGF).^29^ Furthermore, HBx upregulated p27 and p21 protein expression, inhibiting the activity of cyclin‐dependent kinase 4 (CDK4), which led to the uncontrolled proliferation of hepatocytes and, ultimately, to HCC. HBx also controlled the calcium level uptake of mitochondria, which in turn led to an upregulated HBV. In this case, HBx interfered with a tyrosine kinase signalling pathway. Furthermore, HBx induced the inhibition of the P13K/AKT apoptotic pathway to prevent apoptosis of host cells, promoting hepatocyte proliferation.^22^

Overall, various functions performed by HBx indicate its indirect role in the progression of cancer. However, evidence showing the mechanism by which HBx interacts with each transcription factor and regulatory protein involved in tumorigenesis is lacking.

### 3.4 Pathology and treatment of HBV‐induced HCC

Acute liver infection is indicated by necroinflammation in the liver. Measures of the transaminase level can indicate the effectiveness of innate immunity in viral clearance.

Studies have shown that cytotoxic T cells (CTLs) are necessary for HBV clearance.^30^ The prevention of hepatocyte attachment to cells depends on adaptive immunity through which specific neutralizing anti‐HB antibodies, are produced. High levels of HBsAg and HBeAb may disrupt the functions of antigen‐presenting cells, such as dendritic cells (DCs), which are critical for activating T cells, leading to a reduced level of T cells in the blood. Migration of viral proteins to the liver (diapedesis) results in the production of proinflammatory cytokines, which causes immunopathogenesis. Long‐term HBV infection without treatment leads to the development of chronic liver diseases, such as cirrhosis and HCC.^3^

Different kinds of treatments target different phases of HBV infection. For example, antiviral medications prevent further viral replication, often to undetectable levels, in cells, thereby preventing the development of chronic hepatitis. The treatment offered depends on the viral load (>2000 UI/mL) and whether the patient is carrying HBV DNA (>20,000). The two treatments being prescribed for HCC include NAs) which inhibit the synthesis of DNA or RNA by blocking the replication process, and PEGylated interferon‐alpha (PegIFN‐α).^31^ Host risk factors associated with HCC are being older than 40 years, consuming high levels of alcohol, being male, taking immunosuppressive drugs or being infected with HIV.

Tenofovir is an NA that inhibits reverse transcription activity by competing with natural nucleotides. Only two phosphorylation steps are needed to induce metabolism. TDF dephosphorylates and competes for the HBV polymerase active site because its binding affinity is higher than that of host human DNA polymerase.^32^ Studies have shown that patients infected with the (lamivudine) LAM‐resistant virus take 300 mg of TDF each day, resulting in a reduction in the copies of HBV DNA/mL serum by a 4–6 log10 magnitude.^33^ Compared with entecavir (ETV), which is a guanosine nucleotide, TDF targets the active site of HBV polymerase. Research has shown that the mortality rate was significantly lower in patients taking TDF (by 16%) than in those taking ETV, indicating that TDF therapy leads to longer recurrence‐free survival than is induced by ETV.^32^ However, the side effects of TDF can be severe. TDF can accumulate in the blood plasma and cause solutes imbalance at the proximal tubules. This leads to a higher rate of TDF uptake and lowers the elimination of TDF in the urine (Wassner, 2020).^34^

PegIFN‐α is a soluble glycosylated cytokine. It is produced in infected host cells, such as plasmacytoid dendritic cells (DCs), with antiviral activity. In early 1990, IFN was used to treat HBV, but in 2005, a new treatment for HBV based on PEGylated IFN‐alpha resulted in more suitable pharmacokinetics and a reduction in the required dosage to once per week.^19^ Twenty‐four weeks of PegIFN‐α treatment resulted in a 24% response rate versus a 12% response rate, as measured by the reduction in HBeAg level.^35^ However, this success rate is lower than that recommended based on 5–10 million units delivered thrice per week. Therefore, the most effective PegIFN‐α treatment dosage remains unclear. IFN‐alpha reduces viral replication by inhibiting the transcription of cccDNA. IFN regulates cccDNA expression by interacting with the H3 and H4 histone proteins mediated via hypoacetylation and corepressor recruitment.^34^

Apolipoprotein B mRNA‐editing enzyme catalytic polypeptide‐like 3 G (APOBEC3G) expression is suppressed during viral replication, similar to its reduced expression after HIV infection.^24^ APOBEC3G increases transcriptional errors. IFN upregulates APOBEC3G expression and thus activates and drives the proliferation of cytotoxic T cells and NK cells to increase the rate of viral clearance.^24^

PegIFN and NAs are the primary recommended treatments for HBV. Nevertheless, they have pros and cons in terms of either safety or efficacy. A benefit of PegIFN is the finite duration of treatment and high rate of seroconversion to produce anti‐HB antibodies. PegIFN does not induce drug resistance. However, IFN can cause increased morbidity and mortality in patients. In contrast, NAs induce fewer adverse effects, and some can be prescribed during pregnancy and taken orally. Newly synthesized TDV and ETV induce little or no drug resistance. The major disadvantage of NAs their low seroconversion rates, which leads to increased treatment duration and results in an unclear timeframe for viral clearance^24^ (Table 2).

**TABLE 2:** An overview of treatments for hepatocellular carcinoma (HCC) associated with hepatitis B virus (HBV).

Nucleotide Analogue (TDF)	PegIFN
Mechanism	
TDF compete with the host cell nucleotides during DNA synthesis. TDF inhibits the reverse transcription activity of HBV polymerase	IFN‐alpha reduces viral replication by inhibiting the transcription of cccDNA. IFN regulates cccDNA expression by interacting with the H3 and H4 histone proteins mediated via hypoacetylation and corepressor recruitment
Adverse effects	
Can cause kidney failure by damage the proximal tubules. This worsens renal impairment	Common adverse effect can be nausea, stomach cramp, diarrhoea.
Reference	
Wassner (2020)^34^	Bamford and Zuckerman^11^

## 4 CONCLUSION

Since 1965, Dr Baruch Blumberg, who won the Nobel Prize for the discovery of HBV, has made a large contribution to HBV infection prevention and treatment, particularly in relation to HBV‐related HCC. Significant decreases in the incidence and mortality rates associated with HCC have been observed worldwide since 2005. This promising indicator suggests the mechanism of viral infection is clearly comprehended and that the drugs invented to date effectively inhibit certain viral replication processes, such as the inhibition of reverse transcription to prevent the integration of dslDNA or transcription. However, the direct mechanism underlying HBV‐induced HCC incidence remains unclear. Moreover, the research focus on HBx protein has explained the majority of the indirect mechanisms by which HBV induces HCC. By further investigating HBx protein function, the development of drugs can be further improved; for example, drugs that inhibit transposon activity, which would reduce the risk of uncontrolled hepatocyte proliferation, can be developed.

## AUTHOR CONTRIBUTIONS

**LITAO ZHENG:** Conceptualization (equal); formal analysis (equal); project administration (equal); resources (equal); software (equal); supervision (equal); validation (equal); writing – original draft (equal); writing – review and editing (equal).

## CONFLICT OF INTEREST STATEMENT

The author confirms that there are no conflicts of interest.

## Data Availability

This dissertation is not updated with the relevant new data. All the analysis is adopted from the existing data, and the reference shows the adopted details.
